# The Dose‐Response Relationship Between Leisure‐Time Physical Activity and Smartphone Addiction in First‐Year Tehran University Students: A Cross‐Sectional Study

**DOI:** 10.1002/hsr2.72581

**Published:** 2026-06-07

**Authors:** Hosein Ataei‐Goujani, Keyvan Karimi, Anis Gharajeh, Sarmad Salehi, Yosef Farzi, Mahnaz Khalafehnilsaz, Samaneh Akbarpour, Yosra Azizpour

**Affiliations:** ^1^ Occupational Sleep Research Center, Baharloo Hospital Tehran University of Medical Sciences Tehran Iran; ^2^ Student Research Committee Shahrekord University of Medical Sciences Shahrekord Iran; ^3^ Non‐Communicable Diseases Research Center, Endocrinology and Metabolism Population Sciences Institute Tehran University of Medical Sciences Tehran Iran; ^4^ Department of Epidemiology and Biostatistics, School of Public Health Tehran University of Medical Sciences Tehran Iran; ^5^ Endocrinology and Metabolism Research Center, Endocrinology and Metabolism Clinical Sciences Institute Tehran University of Medical Sciences Tehran Iran; ^6^ School of Health and Society, Faculty of Education Health and Wellbeing University of Wolverhampton Wolverhampton UK; ^7^ Sleep Breathing Disorders Research Center Tehran University of Medical Sciences Tehran Iran

**Keywords:** dose‐response, physical activity, smartphone addiction, students

## Abstract

**Background and Aims:**

Smartphone addiction (SA) is highly prevalent among first‐year university students and has significant consequences. Physical activity (PA), known to benefit both physical and mental health, has shown inconsistent associations with SA across studies. Moreover, the distinct sociocultural context of Iran with respect to PA and SA suggests that these associations may differ from those observed in other populations. Given these inconsistencies, this study aimed to examine the relationship between PA and SA among first‐year students at Tehran University of Medical Sciences (TUMS).

**Methods:**

In this cross‐sectional study, baseline data from 397 first‐year TUMS students were analyzed. SA was measured using the Smartphone Addiction Scale‐Short Version (SAS‐SV) and PA with the Global Physical Activity Questionnaire (GPAQ‐v2). Associations were examined using multivariable linear regression and dose‐response analysis.

**Results:**

Participants engaged in sufficient moderate leisure‐time physical activity (LTPA) had the strongest inverse association with SA (adjusted *β* = −5.04, 95% CI: −8.57 to −1.52, *p* = 0.005) compared to other PA domains. In addition, trend analysis revealed that LTPA exhibited a significant trend in the level of PA and severity of SA. Additionally, the dose‐response analysis revealed that higher PA levels were associated with lower levels of SA, with this relationship being most pronounced at lower PA amounts.

**Conclusion:**

The current association between LTPA and SA could suggest that promoting LTPA may be particularly effective in mitigating smartphone addiction among first‐year university students.

**Abbreviations**
CIs: confidence intervals
GPAQ‐v2: second version of Global Physical Activity Questionnaire
LTPA: leisure‐time physical activity
MET: metabolic equivalent task
NCD: noncommunicable disease
PA: physical activity
SA: smartphone addiction
SAS‐SV: Smartphone Addiction Scale‐Short Version
SD: standard deviation
SES: socioeconomic status
STEPS: STEPwise Approach to NCD Risk Factor Surveillance
STROBE: Strengthening the Reporting of Observational studies in Epidemiology
TUMS: Tehran University of Medical Sciences
WHO: World Health Organization

## 1 Introduction

Smartphone addiction (SA) is a behavioral dependency characterized by significant physical and psychological symptoms, including withdrawal and loss of control, which interferes with daily life [1]. Despite extensive research, a valid consensus on the term referred to smartphone overuse remains inconsistent across studies, and different studies named it differently, such as ‘smartphone dependency,’ ‘problematic smartphone use,’ and ‘compulsive phone use’ [2, 3]. However, although terminology varies, the core features such as loss of control, withdrawal, and interference with daily life are consistent across studies [2, 4, 5]. SA is rising globally and has been described as a public health concern [6, 7]; alarmingly, university students are particularly vulnerable to this issue [8]. Among these, the prevalence of SA in first‐year university students seems to be substantially higher [9]. Meta‐analysis estimated SA prevalence around 41.93% among Asian medical students [10], with similarly high prevalence—46.6% and 71.3%—among Iranian students [11, 12].

According to the WHO, physical activity (PA) is defined as “any bodily movement produced by skeletal muscles that requires energy expenditure” [13]. PA not only yields significant physical health advantages but also contributes positively to mental health outcomes [14, 15]. Nonetheless, in spite of these well‐documented benefits, Iran's national data—STEPwise Approach to noncommunicable disease (NCD) Risk Factor Surveillance (STEPS)—demonstrates increasing physical inactivity among young adults in Iran, paralleling global trends [16, 17]. Despite the STEPS national studies, specific data on the PA pattern among university students remained heterogenous and limited. Existing studies have reported the prevalence of physical inactivity in Iran's university student population from approximately 50% [18] to 73% [19]. However, PA pattern in the first year, specifically, are less explored. In addition, leisure‐time (recreational) physical activity (LTPA) has recently attracted attention due to its crucial and more prominent effect on physical health, probably through social interaction [20, 21, 22]. As an example, in a study on depressive symptoms, unlike work, household, or transportation PA, LTPA has been consistently linked with protective effects, including reduced depressive symptoms [23]. In addition, multiple studies have demonstrated a negative association between PA and SA [24, 25]. However, despite the importance of LTPA, Iran's economic fluctuations and unorganized development in technology and the entertainment industry has influenced LTPA levels [26], as a nationwide study in Iran reported that physical inactivity is most prevalent in the LTPA domain, with an age‐standardized prevalence of 79.40% (95% confidence interval [CI]: 78.80–79.99), compared with the work‐related and transport‐related PA domains [17].

Despite evidence of an inverse PA–SA association, findings remain inconsistent among university students [24, 25]. As discussed, the prevalence of SA exhibited significant variation across studies involving Iranian students and warrants further investigation, particularly among first‐year students. Moreover, the sociocultural context of Iranian younger population is particularly distinctive with respect to SA and PA. Previous research underscores the role of cultural and family context in shaping SA. Iranian studies suggest that students’ dependence on smartphones varies across families with varying socioeconomic and cultural backgrounds [27]. In addition, cross national evidence indicates that cultural orientation may be relevant to addictive smartphone related behaviors. A meta‐analysis across 32 nations reported a substantially higher prevalence of addictive social media use in collectivist cultures at 31% compared with 14% in individualist cultures [28]. Iranian family and gender norms may further shape smartphone related behaviors, such that families may exert greater control over their daughters’ smartphone use, while boys tend to show a greater interest in digital gaming on smartphones from younger ages [29]. Beyond smartphone use, collectivist leaning contexts may also influence LTPA levels compared to individualist cultural dimension [30, 31]. Moreover, within the Iranian cultural context, females aged 18–24 years have been reported to exhibit a substantially higher prevalence of physical inactivity compared with their male counterparts [17]. Taken together, given the unique sociocultural context of Iranian university students and complex, not fully understood relationship between PA, specifically LTPA, and SA among Iranian students, further research is warranted, especially in first‐year students due to the high prevalence of SA and its substantial consequences [9, 32].

We designed a study aimed to examine the relationship between PA and SA specifically focousing on Iranian sociocultural context among first‐year students at Tehran University of Medical Sciences (TUMS), with a particular focus on dose‐response patterns and the role of LTPA.

## 2 Methods

### 2.1 Study Design, Participants, and Data Source

This study was a cross‐sectional secondary analysis of baseline data from the TUMS cohort on lifestyle and NCD risk factors among first‐year students [33]. In the current study, all participants enrolled voluntarily, and those who were 18 years old or older and willing to participate were enrolled. In contrast, non‐Iranian first‐year students were not included in the study. We utilized data from the Phase 1 (baseline) cohort study of lifestyle and NCD risk factors conducted at TUMS from December 4, 2024, to February 19, 2025 [33]. In this phase we used data related to questionnaires regarding students’ demographics, lifestyle, and mental health status. This observational study was reported according to the Strengthening the Reporting of Observational Studies in Epidemiology (STROBE) guideline [34].

### 2.2 Definition of Variables

#### 2.2.1 Smartphone Addiction Scale‐Short Version (SAS‐SV)

SA was assessed using the SAS‐SV tool [35], and its reliability and validity have been previously assessed among Iranian students with Cronbach's *α* equal to 0.85 [36]. Possible scores range from 10 to 60, and higher scores represent more severe addiction.

#### 2.2.2 Second Version Global Physical Activity Questionnaire (GPAQ‐v2)

PA level was assessed using the second version of the Global PA Questionnaire (GPAQ‐v2) tool with little modification [37, 38]. This tool was developed and validated by WHO to measure PA level and has been previously validated in Iran. This tool assesses PA in three domains of everyday life, including work, transport, and LTPA, alongside sedentary behavior during a week. Additionally, LTPA and work PA are categorized into moderate and intensive PA, while transport PA only assesses moderate PA. However, we eliminated work‐related questions to reduce question time due to their lower implication in university students’ lives [39, 40]. As summarized in Supporting Information S1: Table S1, participants were classified into three PA categories according to the total weekly duration of moderate and intensive activity, in accordance with WHO guideline [38].

### 2.3 Covariates

In this study, covariates were selected based on the previous literature review. Demographic variables including age and gender [41], socioeconomic status (SES)—categorized into tertiles [42], and living arrangement—categorized into dormitory and home [43]. Dietary habits were assessed using a constructed dietary score derived from self‐reported mean daily intake of five macronutrient groups—fruits, vegetables, protein, carbohydrates, and dairy products. The sum of the mean daily units of these components was used to generate a composite score representing overall dietary healthiness, which was subsequently categorized into tertiles [44, 45]. Sleep duration was assessed by asking each participant's night sleep duration (hours/night)—categorized into normal (7–9 h/day) and abnormal [46, 47]—and alcohol consumption [48] and smoking [49] usage were considered as considerable potential covariates.

### 2.4 Statistical Analysis

We analyzed the current study results utilizing STATA 17. Categorical variables were presented as frequency (percentage) and were analyzed with the chi‐square or Fisher's exact test when each was suitable. Continuous variables were presented as mean ± standard deviation (SD) and were compared using an ANOVA test. Prior to the conduction of the linear regression analysis, the normality of residuals was assessed, and if the SAS‐SV was distributed non‐normally, suitable transformations were conducted for further analysis. The univariable and multivariable regression was applied to reveal crude and adjusted associations of PA with SA. Adjusted models included age, sex, SES, living arrangement, diet, sleep duration, smoking, and alcohol consumption for potential confounding. Additionally, based on Green's rule‐of‐thumb for multiple linear regression—which recommends a minimum of 10–20 participants per predictor variable—our sample size was deemed sufficient to ensure reliable regression estimates [50]. The results were presented as beta coefficients and their corresponding 95% CIs. The dose‐response trends of SA changes with different levels of PA for each domain based on the metabolic equivalent task (MET) score [17] were assessed using a restricted cubic spline function and assuming four knots (quartiles) [51]. The Y‐axis on this graph is specifically the predicted SA score [52]. After excluding missing values in the outcome and primary exposure, other analyses were performed using pairwise deletion in the univariable analysis and complete‐case deletion in the final regression model. To assess potential selection bias due to missing data, we first compared the distribution of basic demographic variables (age and sex) between participants with complete GPAQ‐V2 and SAS‐SV data and those with missing data. The age and sex distributions did not differ considerably in the two groups. Since the missing‐data pattern in our study was random and lacked a specific pattern, we conducted a sensitivity analysis using imputed values for missing data. The final results did not change significantly compared to the results obtained from the deleted missing data. For all the analyses, a two‐sided test was conducted, and a *p* value < 0.05 was considered statistically significant.

## 3 Results

### 3.1 Descriptive of Study Participants

**Figure 1:**
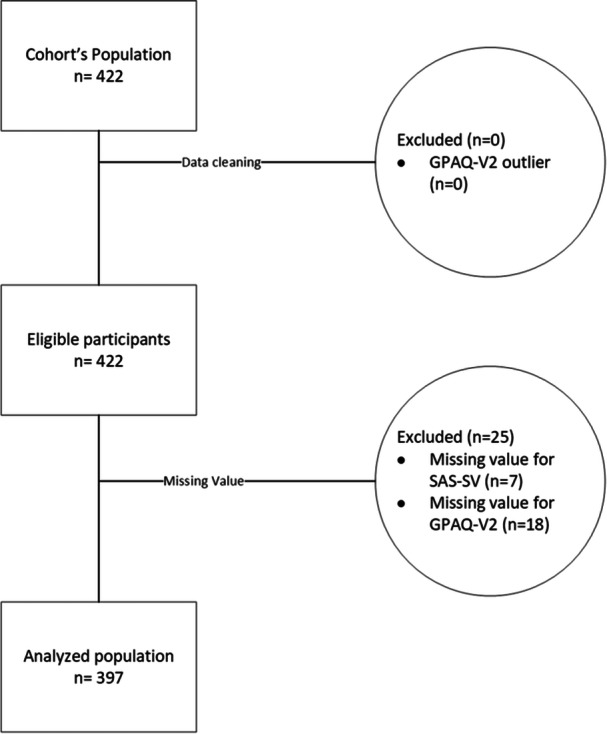
Study flowchart of study participants. Abbreviations: GPAQ‐V2, second version of the Global Physical Activity Questionnaire; SAS‐SV, Smartphone Addiction Scale‐Short Version.

A total of 422 first‐year university students were enrolled in the primary study, as shown in Figure 1. Out of this population, a total of 25 students (5.9%) were excluded due to missing data on the GPAQ‐V2 questionnaire (*n* = 18) or the SAS‐SV questionnaire (*n* = 7), resulting in a total of 397 students being analyzed.

### 3.2 Participant's Demographics and Lifestyle Characteristics

Table 1 represents participants’ demographics and lifestyle characteristics based on different levels of PA. Participants’ population aged 19.5 ± 3.4; most of them were female (54%), lived at home (56%), and reported abnormal sleep (62%). Moreover, a comparison of participants’ mean ages across tertiles of PA levels utilizing One‐way ANOVA exhibited that inactive participants had a mean age of 19.8 ± 3.4 years, insufficiently active participants 20.3 ± 6.2 years, and sufficiently active participants 19.2 ± 1.9 years, with no statistically significant differences among the groups (*F* value = 2.85, *p* = 0.052). Additionally, with respect to dietary habits, comparison across PA tertiles using the Chi‐square test revealed that sufficiently active participants had a lower proportion of the lowest diet habit tertile (70 [28.69%]) compared to insufficiently active (33 [44%]) and inactive participants (21 [46.67%]) (Chi^2^ = 9.07, *p* = 0.059).

**Table 1:** Descriptive features of student based on physical activity levels.

Variables^a^	Total population	Physical activity levels no (%)			Chi^2^	*p* value
		Inactive 48 (12.1)	Insufficient 82 (20.6)	Sufficient 267 (67.3)		
**Sex**—no [%]						
Female	213 [53.65]	31 [64.58]	49 [59.76]	133 [49.81]	4.99	0.082
Male	184 [46.35]	17 [35.42]	33 [40.24]	134 [50.19]		
**Living arrangement**— no [%]						
Dormitory	175 [44.08]	27 [56.25]	32 [39.02]	116 [43.45]	4.07	0.138
Home	222 [55.92]	21 [43.75]	50 [60.98]	151 [56.55]		
**Socioeconomic status** b— no [%]						
Low	55 [13.89]	7 [14.89]	14 [17.07]	34 [12.73]		
Average	234 [59.09]	29 [61.7]	50 [60.98]	155 [58.05]	2.79	0.593
High	107 [27.02]	11 [23.4]	18 [21.95]	78 [29.21]		
**Diet** c— no [%]						
Low	124 [34.07]	21 [46.67]	33 [44.00]	70 [28.69]		
Moderate	121 [33.24]	10 [22.22]	22 [29.33]	89 [36.48]	9.07	0.059
Good	119 [32.69]	14 [31.11]	20 [26.67]	85 [34.84]		
**Alcohol consumption**—no [%]						
No	377 [95.44]	44 [91.67]	80 [98.77]	253 [95.11]	3.86	0.145
Yes	18 [4.56]	4 [8.33]	1 [1.23]	13 [4.89]		
**Smoking**— no [%]						
No	358 [90.63]	41 [85.42]	73 [90.12]	244 [91.73]	1.10	0.574
Yes	37 [9.37]	7 [14.58]	8 [9.88]	22 [8.27]		
**Secondhand smoking**—no [%]						
No	139 [35.19]	20 [41.67]	35 [43.21]	84 [31.58]	4.76	0.092
Yes	256 [64.81]	28 [58.33]	46 [56.79]	182 [68.42]		
**Sleep duration** d—no [%]						
Abnormal sleep	243 [61.68]	29 [60.42]	53 [64.63]	161 [60.98]	0.40	0.815
Normal sleep	151 [38.32]	19 [39.58]	29 [35.37]	103 [39.02]		

*Note:* Data are presented as number [columnar %]. All variables were compared using Spearman's Chi‐square test.

Abbreviations: no, number; SD, standard deviation.

^a^ Missing data for each variable: Socioeconomic status: *n* = 1, diet: *n* = 33, alcohol consumption: *n* = 2, second‐hand smoking: *n* = 2, sleep duration: *n* = 3.

^b^ Categorized into tertiles.

^c^ Consumption of fruit, vegetables, protein, carbohydrate and dairy (units per day), tertile the sum score of these five food groups.

^d^ Categorized into normal (7–9 h/day) and abnormal.

### 3.3 Pattern of PA Across SA

As Spearman's Chi‐square test indicates, for both Total PA and Travel‐related PA, the proportions across activity categories (Inactive, Insufficient, Sufficient) were similar and statistically non‐significant (*p* = 0.910 and *p* = 0.869, respectively). However, moderate LTPA revealed statistically significant differences (*p* = 0.018), with a higher proportion of inactive individuals and a lower proportion of sufficiently active individuals in the high‐SA group compared with the low‐SA group. A similar, though non‐significant (*p* = 0.103), pattern was observed for intensive LTPA (Table 2).

**Table 2:** Pattern of physical activity among students based on smartphone addiction tertiles.

Variable	Smartphone addiction (tertiles)			Chi^2^	*p* value
	Low	Moderate	High		
**Total PA** a—no [%]					
Inactive	14 [10.45]	16 [12.03]	18 [13.85]		
Insufficient	27 [20.15]	26 [19.55]	29 [22.31]	0.998	0.910
Sufficient	93 [69.4]	91 [68.42]	83 [63.85]		
**Travel** a—no [%]					
Inactive	24 [17.91]	27 [20.3]	24 [18.46]		
Insufficient	70 [52.24]	61 [45.86]	66 [50.77]	1.25	0.869
Sufficient	40 [29.85]	45 [33.83]	40 [30.77]		
**Moderate LTPA** a—no [%]					
Inactive	65 [48.51]	78 [58.65]	89 [68.46]		
Insufficient	17 [12.69]	9 [6.77]	9 [6.92]	11.88	**0.018**
Sufficient	52 [38.81]	46 [34.59]	32 [24.62]		
**Intensive LTPA** a—no [%]					
Inactive	71 [52.99]	78 [58.65]	90 [69.23]		
Insufficient	26 [19.4]	22 [16.54]	19 [14.62]	7.70	0.103
Sufficient	37 [27.61]	33 [24.81]	21 [16.15]		

*Note:* Data are presented as number (columnar %) and were compared using Spearman's Chi‐square test. Bold values represent statistical significance.

Abbreviations: LTPA, leisure time physical activity; no, number; PA, physical activity.

a Classification of participants based on weekly physical activity level: Inactive (no moderate or intensive activity), insufficiently active (< 150 min of moderate activity, < 75 min of intensive activity, or equivalent combination < 150 moderate‐equivalent minutes), and sufficiently active (≥ 150 min of moderate activity, ≥ 75 min of intensive activity, or an equivalent combination of ≥ 150 moderate‐equivalent minutes; 1 min of intensive activity = 2 min of moderate activity).

### 3.4 The Relationship Between SA and PA

**Figure 2:**
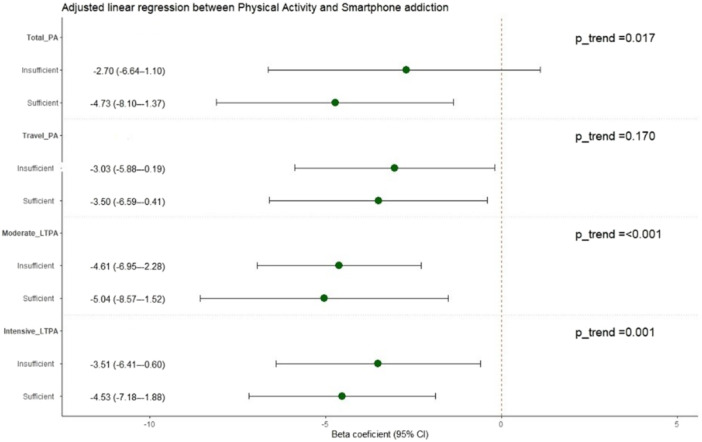
Forest plot of the association between physical activity and mobile addiction score results from the regression model, adjusted for age, sex, SES, alcohol consumption, tobacco use, second‐hand smoking, and diet. Abbreviation: LTPA, leisure‐time Physical activity.

A multivariable linear regression analysis was used to examine the relationship between PA and SA. The results revealed that participants engaged in sufficient moderate LTPA had the strongest inverse association with SA (adjusted *β* = −5.04, 95% CI: −8.57 to −1.52, *p* = 0.005). In addition, univariable regression analysis examining the association between total PA and SA showed that participants with sufficient total PA had significantly lower SA scores in the crude model (*β* = −4.39, 95% CI: −7.67 to −1.11, *p* = 0.009). This association persisted as significant and became slightly stronger after adjusting for covariates including age, sex, SES, living arrangement, sleep quality, second‐hand smoke exposure, and risky behaviors (adjusted *β* = −4.73, 95% CI: −8.01 to −1.37, *p* = 0.006). Despite the presence of an inverse association of insufficient PA, the association did not reach statistical significance in either model. Moreover, the estimates from the imputed analyses were comparable to those from the complete‐case analyses, suggesting that results were robust (Figure 2 and Table 3).

**Table 3:** The relationship between smartphone addiction and physical activity in crude and adjusted models.

	Crude model		Adjusted model^a^	
Total PA	*β* [95% CI]	*p* value	*β* [95% CI]	*p* value
Inactive	Ref b		Ref	
Insufficient	−3.34 [−7.14,0.45]	0.084	−2.70 [−6.64, 1.10]	0.160
Sufficient	−4.39 [−7.67, −1.11]	**0.009**	−4.73 [−8.01, −1.37]	**0.006**
Trend	−1.92 [−3.42, −0.42]	**0.020**	−1.89 [−3.50, −0.29]	**0.017**
**Travel**				
Inactive	Ref		Ref	
Insufficient	−2.32 [−5.18, 0.52]	0.109	−3.03 [−5.88, −0.19]	**0.036**
Sufficient	−2.36 [−5.43, 0.70]	0.130	−3.50 [−6.59, −0.41]	**0.027**
Trend	−1.03 [−2.54, 0.47]	0.173	−1.07 [−2.6, 0.54]	0.170
**Moderate LTPA**				
Inactive	Ref		Ref	
Insufficient	−4.37 [−6.67, −2.08]	**< 0.001**	−4.61 [−6.95, −2.28]	**< 0.001**
Sufficient	−4.64 [−8.14, −1.14]	**0.009**	−5.04 [−8.57, −1.52]	**0.005**
Trend	−3.00 [−4.58, −1.46]	**< 0.001**	−3.25 [−4.90, −1.61]	**< 0.001**
**Intensive LTPA**				
Inactive	Ref		Ref	
Insufficient	−3.37 [−6.21, −0.50]	**0.021**	−3.51 [−6.41, −0.60]	**0.018**
sufficient	−4.29 [−6.85, −1.75]	**0.001**	−4.53 [−7.18, −1.88]	**0.002**
Trend	−2.26 [−3.51, −1.00]	**< 0.001**	−2.22 [−3.59, −0.86]	**0.001**

*Note:* Classification of participants based on weekly physical activity level: Inactive (less than 2 min or no moderate or intensive activity), Insufficiently active (< 150 min of moderate activity, < 75 min of intensive activity, or equivalent combination < 150 moderate‐equivalent minutes), and Sufficiently active (≥ 150 min of moderate activity, ≥ 75 min of intensive activity, or equivalent combination ≥ 150 moderate‐equivalent minutes; 1 min of intensive activity = 2 min of moderate activity). Bold values represent statistically significance.

Abbreviations: LTPA, leisure time physical activity; PA, physical activity.

^a^ Adjusted for age, sex, SES, living arrangement (dormitory or home), normal sleep, second‐hand smoking, risky behavior (smoking and alcohol use).

^b^ The reference group.

In addition, trend analysis revealed that higher PA levels were consistently associated with lower SA scores, especially for LTPA. Specifically, in total PA and LTPA domains, in contrast to the travel domain, exhibited significant trends in the level of PA and severity of SA. Likewise, the strongest inverse trend was observed between moderated LTPA and SA among first‐year university students (adjusted *β* for trend = −3.25, 95% CI: −4.90 to −1.61, *p* < 0.001) compared to another PA domain (Figure 2 and Table 3).

### 3.5 The Dose‐Response Association Between SA and PA

**Figure 3:**
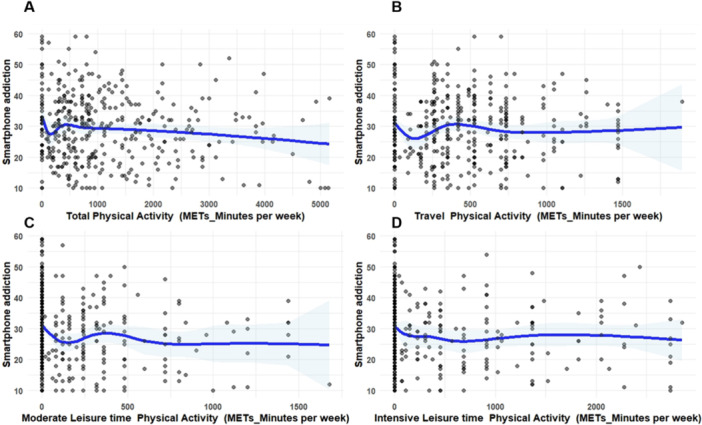
The dose‐response association between PA levels based on MET‐minutes per week and SA score modeled on a cubic spline prediction with four knots. Associations are shown for (A) total PA, (B) travel PA, (C) moderate LTPA, (D) intensive LTPA. The solid blue line indicates the predicted smartphone addiction score, and the gray shaded area represents 95% CI. Abbreviation: MET, metabolic equivalent task.

As illustrated in Figure 3, the dose‐response curves, a distinct inverse relationship was observed between various domains of PA and predicted SA scores. Among the four types of PA—total PA, transport‐related PA, moderate‐LTPA, and intensity LTPA—the most significant reduction in predicted addiction scores was noted at lower levels of activity (i.e., lower MET‐min/week). However, a plateau association was observed at higher PA levels, suggesting a weaker inverse association.

## 4 Discussion

This study found an inverse dose‐response association between PA and SA, with LTPA showing the strongest association. Likewise, the current study aligns with previous similar studies, according to a recent systematic review of literature, although a substantial number (25/28) of previous studies reported a similar inverse association of PA and SA; however, the magnitude of this association varied considerably, ranging from negligible to strong effects, indicating substantial heterogeneity across findings [29, 53]. Furthermore, our study showed that even an insufficient amount of LTPA compared to complete inactivity is considerable, emphasizing that even a sub‐effective amount of LTPA could be substantial. Specifically, merely as little as 160 MET/week, equals to 40 min/week engagement in moderate LTPA, can be associated with considerably lower SA. This stronger association may address observed heterogenicity between studies. LTPA has been considered as a unique domain of PA due to its distinct influence on a range of various health‐related outcomes, beyond what is seen in other domains [54]. Notably, it has been discussed that LTPA even may exert a contrast effect compared to other domains [55]. In addition to the observed inverse association of LTPA and SA, LTPA may also serve as a modulator for alleviating negative consequences of SA. Specifically, a recent study found that individuals with high SA levels who engaged in greater LTPA experienced lower levels of loneliness [56].

Among first‐year university students, the stronger association between LTPA and reduced SA may be explained by self‐determined participation. LTPA often involves self‐selected intensity and voluntary engagement—a component that we may not see in other domains. One potential hypothesis for this stronger association is that LTPA's self‐determined nature may engage neural pathways involved in self‐control, such as those mediated by the prefrontal cortex [57]. Although these cognitive functions are critical in managing behavioral addictions such as SA [58, 59], this would require direct investigation.

Despite the observed association between higher LTPA and lower SA, Iran has a considerable high prevalence of physical inactivity in LTPA domain with about 80% which specifically is concerning among women [17], which could be as a result of economic fluctuations [26] and gender‐speific sociocultural norm including sociocultural expectations, limited family support, and reduced access to supportive environments for PA [60]. Additionally, structural determinants such as economic inequality, urbanization, and lifestyle transitions have been shown to further exacerbate physical inactivity patterns in Iran [61].

On the other hand, from a cross‐cultural perspective, evidence from collectivist‐dominant societies such as China suggests that psychosocial dynamics such as need for individuality may drown students into mobile phone usage which in such contexts, digital engagement may function as a compensatory mechanism [62]. Given that Iran similarly reflects collectivist cultural characteristics, the growing prevalence of SA among students may be partially understood within this socio‐cultural framework.

Importantly, PA behaviors in collectivist settings are strongly influenced by social support and group dynamics [63]. In this regard, the strong association observed between LTPA and SA in the present study may reflect the socially embedded nature of leisure‐time PA in Iran specific cultural context. Therefore, interventions aimed at enhancing social support and promoting group‐based LTPA may represent a culturally appropriate strategy for reducing SA, warranting further investigation in future studies.

Our dose‐response analysis strengthens the plausibility of an inverse relationship, though causality cannot be established given the cross‐sectional study design limits the ability to infer causality. Furthermore, since clinical trials have demonstrated a positive effectiveness of different PAs on reducing SA [64], future interventional studies could specifically investigate the effectiveness of interventions on promoting voluntary LTPA on SA and compare its effectiveness with other PA modalities to further improve our understanding of the causal relationship of LTPA with SA. Importantly, given the importance of SA among first‐year students, our study findings may provide preliminary evidence to inform future policy considerations and university health initiatives to explore LTPA as a potential strategy for mitigating SA and its related consequences, pending confirmation from longitudinal and interventional research.

Beyond the strengths of our study—including adjusting for different sets of covariates based on a literature review to control confounding bias, the assurance of an adequate sample size providing 74%–85% statistical power across all analyses, and specific sociocultural context of the current studies compared to studies from other nations—several limitations should be acknowledged. Although the primary study was implemented under close supervision with trained interviewers, the self‐reported nature of assessment tools is subject to recall bias and social desirability effects, and participants may unintentionally overestimate their activity levels or underreport screen time, thereby affecting data validity. To address this limitation, future studies can utilize a combination of subjective methods (like questionnaires) with objective tools such as wearable fitness trackers for PA or apps to record actual PA and smartphone usage, respectively. Besides, although we have focused on the first‐year university students at one medical university due to their heightened vulnerability to SA and related consequences, further research is needed to assess similar associations in other high‐risk groups such as schoolchildren. The focus on a single Iranian university population of predominantly medical students may also limit the generalizability of our findings to broader or more diverse groups. Moreover, despite the fact that some previous studies, along with our study, on university students’ studies did not consider the work domain since most of the students did not have any specific job [39, 40], omitting the work domain even among university students may reduce validity in assessing work‐related activities. Additionally, as discussed, the limitation of the current cross‐sectional study design in causal interpretation and inferring directionality could be minimized with longitudinal observational or interventional studies. Finally, there remains a considerable knowledge gap regarding psychobiological mechanisms underlying LTPA's stronger association compared to other PA domains [64]. Addressing this gap should be a priority in future investigations.

## 5 Conclusion

In summary, higher levels of PA were associated with lower SA, with LTPA showing the strongest effect among first‐year university students in the Iranian cultural context. Importantly, given the growing concern of SA among first‐year students, our findings offer preliminary evidence to guide policy and university health initiatives. Promoting LTPA may serve as a potential strategy to reduce SA; however, given the cross‐sectional limitations of the study, further research should assess the LTPA–SA relationship in longitudinal and interventional designs to confirm its efficacy.

## Author Contributions

H.A.G., and K.K., contributed to the conceptualization, methodology, formal analysis, data curation, visualization, writing – original draft, and writing – review and editing. A.G., S.S., Y.F., and M.K. contributed to conceptualization, visualization, and writing – review and editing. S.A. and Y.A. contributed to conceptualization, methodology, data curation, supervision, visualization, and writing – review and editing. All authors read and approved the final manuscript.

## Funding

The authors have nothing to report.

## Ethics Statement

The primary study was conducted in accordance with the Declaration of Helsinki. Additionally, the study protocol was approved by the Research Ethics Committees of Endocrine & Metabolism Research Institute—Tehran University of Medical Sciences with ethics approval code (IR.TUMS.EMRI.REC.1403.112).

## Consent

All participants provided informed consent prior to participation. Participation was voluntary, and participants were assured of anonymity and confidentiality of their responses.

## Conflicts of Interest

The authors declare no conflicts of interest.

## Transparency Statement

Yosra Azizpour affirms that this manuscript is an honest, accurate, and transparent account of the study being reported; that no important aspects of the study have been omitted; and that any discrepancies from the study as planned (and, if relevant, registered) have been explained. All authors have read and approved the final version of the manuscript. Yosra Azizpour and Samaneh Akbarpour had full access to all data in the study and take full responsibility for the integrity of the data and the accuracy of the data analysis.

## Supporting information


**Supporting File 1:** hsr272581‐sup‐0001‐Supplemantary_materials_3.docx.

## Data Availability

The data that support the findings of this study are available from the corresponding author upon reasonable request. The datasets generated and analyzed during the current study are available from the corresponding author upon reasonable request. Due to privacy and ethical considerations related to student information, the data are not publicly accessible. However, de‐identified datasets can be provided to qualified researchers to support verification of the study findings, subject to reasonable request.
