# Supplementary material for: The Dose‐Response Relationship Between Leisure‐Time Physical Activity and Smartphone Addiction in First‐Year Tehran University Students: A Cross‐Sectional Study
Source: Health Sci Rep. 2026 Jun 7;9(6):e72581. doi: 10.1002/hsr2.72581 (PMC13242693; doi:10.1002/hsr2.72581)
Supplement: Supplementary file 1 — Supporting File 1: hsr272581‐sup‐0001‐Supplemantary_materials_3.docx. [file HSR2-9-e72581-s001.docx]

**Supplementary Table S1.** Classification of Physical Activity Levels (1).

| Category | Definition / Criteria |
| --- | --- |
| Physically inactive | Reported no engagement in either moderate- or intensive-intensity physical activity during the week. |
| Insufficiently active | Engaged in <150 minutes of moderate-intensity physical activity per week, or <75 minutes of intensive-intensity physical activity, or a combined total <150 moderate-equivalent^*^ minutes per week. |
| Sufficiently active | Engaged in ≥150 minutes of moderate-intensity physical activity per week, or ≥75 minutes of intensive-intensity physical activity, or a combined total ≥150 moderate-equivalent^*^ minutes per week. |

*1 minute of intensive activity = 2 minutes of moderate activity

1. Organization. WH. Global Physical Activity Questionnaire (GPAQ), Analysis Guide.; 2021.
